# Globalization of Stem Cell Science: An Examination of Current and Past Collaborative Research Networks

**DOI:** 10.1371/journal.pone.0073598

**Published:** 2013-09-12

**Authors:** Jingyuan Luo, Kirstin R. W. Matthews

**Affiliations:** 1 Department of Law, London School of Economics and Political Science, London, United Kingdom; 2 Science and Technology Policy Program, James A. Baker III Institute for Public Policy, Rice University, Houston, Texas, United States of America; The Centre for Research and Technology, Hellas, Greece

## Abstract

Science and engineering research has becoming an increasingly international phenomenon. Traditional bibliometric studies have not captured the evolution of collaborative partnerships between countries, particularly in emerging technologies such as stem cell science, in which an immense amount of investment has been made in the past decade. Analyzing over 2,800 articles from the top journals that include stem cell research in their publications, this study demonstrates the globalization of stem cell science. From 2000 to 2010, international collaborations increased from 20.9% to 36% of all stem cell publications analyzed. The United States remains the most prolific and the most dominant country in the field in terms of publications in high impact journals. But Asian countries, particularly China are steadily gaining ground. Exhibiting the largest relative growth, the percent of Chinese-authored stem cell papers grew more than ten-fold, while the percent of Chinese-authored international papers increased over seven times from 2000 to 2010. And while the percent of total stem cell publications exhibited modest growth for European countries, the percent of international publications increased more substantially, particularly in the United Kingdom. Overall, the data indicated that traditional networks of collaboration extant in 2000 still predominate in stem cell science. Although more nations are becoming involved in international collaborations and undertaking stem cell research, many of these efforts, with the exception of those in certain Asian countries, have yet to translate into publications in high impact journals.

## Introduction

Science and engineering (S&E) research is becoming increasingly globalized. Advances in communication and technology now permit the scientific community to share data and publications within minutes. According to the US National Science and Engineering Indicators, from 1995 to 2010, the number of internationally co-authored publications in the physical, natural, and social sciences more than doubled from 79,128 to 185,303 publications [1].

Previous scholarship demonstrated that multi-institutional collaborations increase citation rates, and publications resulting from international collaborations garner twice as many citations as those produced by scientists working at a single institution or within a single country [2]–[5]. This is also true within the subfield of stem cell research [6]–[7]. There are many possible explanations for this phenomenon. Collaboration can be beneficial through the sharing of resources, ideas, and expertise. It also provides younger researchers and labs more exposure in the international arena, affording them better networking opportunities within the research community.

When the overall S&E publications were reviewed, 43% of internationally co-authored publications involved US-based researchers in 2010, while researchers from Germany and the United Kingdom were second and third with 19% each [1]. This is congruent with the strong S&E cultures in these three countries.

Historically, factors such as geographic proximity, availability of funding for research, language, and even cultural practices relating to work, research, and data-sharing ethics, have played prominent roles in a researcher’s decision to collaborate [8]–[10]. Shared histories, such as the colonial connection between the United Kingdom and the United States, as well as membership in supranational organizations like the European Union, have also influenced the decision to collaborate. Moreover similar national legal infrastructures pertaining to intellectual property rights, similar political ideologies, and educational exchange/scholarship programs may also impact one’s decision to enter into collaborations. The worldwide research environment, however, is changing. S&E expenditures have increased an average of seven percent annually over the past decade [11]. Much of the growth is coming from China, India, and other developing nations and their governments, which are placing increased emphasis on S&E research to help improve economic growth, employment, and social well-being.

The effects of emerging nations’ presence in the international research environment, however, still remain to be examined. Traditional bibliometric analyses have evaluated research trends by country, institute, journal, and field of study [12]. Recently, more attention has been shifted towards studying networks of co-authorship and their effects on publication citation rates [13], [14]. No study, however, has captured the evolution of collaborative partnerships between countries and the changing research networks worldwide. This study, thus, seeks to examine the extant research networks in S&E and how they have developed in the past decade using stem cell science as the area of study.

Stem cell science is a particularly interesting field for a study of international collaborations. Stem cells are undifferentiated cells with the capability for self-renewal and can give rise to a vast array of tissue or organ-specific cells [15]. There are primarily three types of stem cells with varying degrees of differentiation potential: adult stem cells (ASCs), embryonic stem cells (ESCs), and induced pluripotent stem cells (iPSCs). They can be isolated from the embryo (blastocyst) five to six days post-fertilization (ESCs) and from several adult organs (ASCs) [14]. The third class, iPSCs, are generated by reverting adult somatic cells back to a state resembling ESCs by turning on genes associated with this embryonic state [16].

Stem cell research – embryonic, adult, and induced pluripotent – has been identified as one of the most important areas in biomedical research today [12]. It is an emerging area of research, exhibiting rapid growth in the number of publications and patents annually. It is also an area with an intrinsic international nature. Laboratories from 50 different countries have published papers in this area in the past five years [17]. Furthermore, various international organizations–such as the International Society for Stem Cell Research (www.isscr.org), the International Stem Cell Forum (www.stem-cell-forum.net), and the International Consortium of Stem Cell Networks (www.stemcellconsortium.org)–have been established to facilitate the exchange of scientific and policy knowledge. These international organizations were founded while the field of stem cell science was young, thus presenting the opportunity to study collaborative partnerships in a field that was characterized, at its nascence, by high levels of international collaborations.

Finally, because of the controversial nature of stem cell science, more specifically research with human ESCs as well as clinical trial safety issues arising from ASC and potential iPSC therapies, research is currently being conducted under varying policy regimes. Many established, leading nations in biomedical research, such as the United States, the United Kingdom, and Germany, have very disparate policies towards human ESC research. US researchers face a policy regime that is constantly under flux. Until President Barack Obama’s executive order in 2009, the federal government only funded projects limited to human ESC lines created before August 2001, the result of President George W. Bush’s policy [18], [19]. In contrast, the United Kingdom employs a more permissive but highly regulated approach towards stem cell research through the Human Fertilisation and Embryology Authority (HFEA), which operates a strict licensing regime for stem cell lines [20]. And while human ESC lines can be derived in both the United States and United Kingdom, Germany’s stem cell research policy bans their production entirely. However, it does allow the importation and use of cell lines created before May 1, 2007 (previously January 1, 2002) [21], [22].

The highly regulated and sometimes restrictive stem cell research environment in these nations is a stark contrast to the permissive policies in many developing nations, such as China, who has recently bolstered their S&E research infrastructure [23]. China permits both the derivation of human ESC lines as well as the use of therapeutic cloning [24]. Additionally, many purported stem cell therapies are currently being offered in Chinese clinics [25]. Stem cell science, thus, presents a unique opportunity not only to study international collaborative research networks, but also the evolution of these networks under disparate policy regimes.

While it is generally accepted that science is becoming increasingly international, little research has been done to examine the exact nature of this globalization. By mapping collaborative partnerships by country, this research sought to compare the current research networks in stem cell science with networks present a decade ago, with the goal of characterizing the globalization of research in this field.

This study analyzed 2,814 publications from the top 50 journals by impact factor in 2000 and 2010 that publish stem cell research. Data revealed that international publications increased from 20.9% (203 of 969) in 2000 to 36% (664 of 1845) in 2010. The United States remains the most prolific and dominant country, with authorship on nearly 60% of all the papers analyzed in both 2000 and 2010. Asian countries, however, are steadily increasingly their shares of articles in top journals. Chinese stem cell publications increased from 0.41% in 2000 to 4.61% in 2010. The data also indicated that traditional networks of collaborations, those that were prominent in 2000 remain so in 2010, despite persistent differences in national stem cell policies and an increased number of countries involved in stem cell research. And although there was evidence of an increasing number of nations becoming involved in international collaborations and undertaking stem cell research, many of these national efforts, with the exception of certain Asian countries, have yet to translate into publications in high impact journals.

## Methods

To map collaborative partnerships in stem cell research, a bibliometric analysis of publications was performed. Publications were retrieved from Thomson Reuters’ Institute of Scientific Information (ISI) Science Citation Index (Web of Science). This database was chosen for its comprehensive collection of peer-reviewed publications and its use in many prior bibliometric studies [12], [26]. It is also the leading source of bibliometric information, as it indexes quality, peer-reviewed journals and has international, multidisciplinary coverage [27].

Only English-language articles were selected so the abstracts of the articles could be reviewed quickly to ensure that each article truly pertained to stem cell research. A preliminary analysis of retrieved ISI publications indicated that well over 95% of the papers published annually are in English, thus allowing the study to capture the majority of collaborative partnerships.

Stem cell research articles from two years 2000 and 2010 were identified by entering the search string: TS = (“stem cell”). Only research articles were selected, filtering out book chapters, proceedings papers, and books. The year 2010 was selected, as it was the most recent full year for which stem cell publications could be retrieved when the study was initiated. The year 2000 was selected because it followed shortly after Dr. James Thomson and his colleagues at the University of Wisconsin-Madison successfully derived the first human ESC line, a major breakthrough in stem cell research and impetus for further research in the area [28]. Additionally, selecting the year 2000 as a time point allowed for the examination of a decade of development in stem cell research. To confirm the years 2000 and 2010 were representative of the general pool of stem cell publications, a preliminary examination of the top 50 countries with the most stem cell publications from 1990–2010 was conducted and yielded no immediate anomalies from either 2000 or 2010.

Running a search with the above parameters in ISI yielded 3,861 stem cell articles for 2000 and 14,974 articles for 2010, a nearly four-fold increase. After identifying the top 50 journals for 2000 and 2010 by impact factors for the respective years, all stem cell related scientific publications from these journals were selected for further analysis (Table S1 and S2). While impact factor is only one of a number of measurements that reflect the quality and eminence of a journal, it is the most commonly used and accepted in academia [29]. Selecting papers only from the top 50 journals could skew the data towards countries with more established traditions of S&E research. However, analyzing publications from top journals ensured that high quality research endeavors were studied, as these journals typically employ rigorous peer-reviewing mechanisms during their publication processes. Moreover, articles published in high impact journals are more likely to be circulated among researchers internationally and possess a greater impact on future research. Analyzing the publications with the most influence on the direction of global research and the development of therapeutics can reveal whether stem cell research has truly globalized. And as these publications are often the result of prominent research networks, the analysis will indicate whether the most influential research networks have evolved as a result of the increasing number of nations participating in stem cell research, or if these networks have remained relatively largely unaltered. The impact factor of journals was also utilized in lieu of other measurements of publication quality such as the citation rate of individual papers because the citation rate for an article is more dynamic, whereas journal impact factors remain relatively constant over time [30]. Admittedly, in a field undergoing rapid growth such as stem cell science, journal impact factors are likely to experience greater changes, which were taken into account during data collection and in its analysis.

The top 50 journals were identified through a comparison of the top 300 Thomson Reuters’s Journal Citation Reports (JCR) for 2000 and 2010 with the corresponding journals for each year in which stem cell articles were published (Table S1 and S2). Of the 14,974 papers located from 2010, 1,845 were analyzed. Of the 3,861 papers identified from 2000, 969 were analyzed.

In order to gauge the extent of any shifts in the proportion of stem cell types used in research and the possible bias this may contribute to the network analysis, keywords for all the papers were analyzed as a crude examination of content change. More exhaustive analysis is needed in the future to precisely quantify any content changes and correlate these changes with potential biases. In total, over 24,000 keywords were analyzed from both 2000 and 2010, the majority of which were unrelated to the specific stem cells types – embryonic, adult, induced pluripotent, and cancer – identified as distinct major areas of stem cell research. Each article had multiple keywords, with no consistency in the format of the keywords, resulting in each type of stem cell being denoted by an array of keywords. For embryonic stem cells, keywords included embryonic stem-cell(s), embryonic stem cell(s), human embryonic stem cell, and human ESC amongst others. Specific keywords were chosen such that they only corresponded to one of the aforementioned four types of stem cells (see Table 1 for the exact terms searched for each type of stem cell). A search for terms connected to stem cell research in general was also conducted as a control. This search encompassed all of the keywords used for the four specific cell types as well as the terms self-renewal, pluripotent(cy), and differentiation, which apply to stem cells in general but cannot be used to identify specific types.

**Table 1:** Stem cell publications keyword analysis.

	2000	2010
	Keywords**	Keywords
**Adult Stem Cells**	8.1%	6.3%
**Embryonic Stem Cells**	1.7%	1.6%
**Induced Pluripotent** **Stem Cells**	0%	0.18%
**Cancer Stem Cells**	3.6%	5.9%
**Stem Cells**	17.0%	19.0%

* The areas of research and their corresponding keywords are as follows: “Adult Stem Cells” (Bone marrow; Marrow; Hematopoietic; Endothelial cell(s); Liver; Umbilical; Cardiomyocyte; Cardiac; Central-nervous-system; Epithelial-cell(s); Erythropoeisis; Hematopoiesis; Hematopoietic-stem; Hematopoietic stem-cell; Neuron; Progenitor),”Embryonic Stem Cells” (Embryo(s); Embryonic; Embryonic stem-cell; Embryonic-development; Embryogenesis; ES-cell(s); Human embryonic stem), “Induced Pluripotent Stem Cells” (IPS Cell(s)), and “Cancer Stem Cells” (Myeloid-Leukemia; Leukemia; Cancer; Chemotherapy: Tumor; Breast cancer; BCR-ABL; Myelogenous; Carcinoma; Chronic-myeloid leukemia; CML).

** Percentages are low due to the large number of keywords (15,526 for 2010 and 9,271 for 2000) analyzed, many of which do not specifically refer to a type of stem cell. The general stem cells category serves as a control, indicating that the percentage of keywords explicitly referring to stem cells is low and remains a relatively constant proportion of total keywords.

15,526 and 9,271 keywords were associated with articles analyzed in 2010 and 2000, respectively. Categories of stem cell research and corresponding keywords were used to analyze any potential shifts in research areas from 2000 to 2010. Specific keywords were chosen such that they only correspond to one type of stem cell.*

There are several methods for counting collaborations, many of which, including the fractional counting method, do not apply well to examining collaborations between countries, as they provide a numerical measurement of collaboration but do not indicate network relationships [9]. For this study, the collaborations were noted, with the country affiliation of the corresponding author(s) demarcated with an asterisk after the country name at the first level of analysis. This is because corresponding authors typically have a greater role in leading and funding the research project, thus a theoretically greater role in the collaboration. From this, the reoccurring two-way, three-way, four-way, etc. collaborations were recorded.

Next, partnerships were examined by recording each publication as a set of pairings. Publications with four countries, for instance, would result in six different pairings. Following this, the tendency of two nations to collaborate was determined by calculating a ratio of the observed to expected co-authorship frequency [10]. The following formula was used to calculate observed to expected ratio (E_r_) for each set of countries:
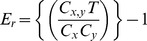
where

C_x_ = total number of collaborations for country X

C_y_ = total number of collaborations for country Y

C_x,y_ = total number of collaborations between country X and country Y

T = total number of collaborations in a group of countries

The value of the ratio, then, is an indication of country X and Y’s propensity to collaborate with each other. The results of the formula depend not only on the two countries for which the ratio is calculated, but also on the presence of the other nations in the group. The ratio, thus, does not describe an exact relationship between country X and Y, rather, it illustrates tendency of the two countries to collaborate within a specific group of nations. In this study, the specific group of nations refers to those (35 for 2000 and 47 for 2010) that participated in international publications. The value used for T was the total number of partnerships in which these nations participated, 426 for 2000 and 1850 for 2010. Two different manners of examining the data were utilized. One examined the prevalent collaborations (two or more countries, one of which is a corresponding author). The second focused on partnerships (two countries, one of which can be but is not necessarily a corresponding author). The resulting data for the two look similar because the top collaborations also happened to be partnerships. For the remainder of the paper, the words collaboration and partnership will be used to help differentiate these two manners of data analysis. All the above patterns were then examined in the context of each nation’s policies towards stem cell research.

## Results

Of the 969 publications analyzed from the year 2000, 203 of the papers (20.9%) were international publications involving two or more countries (Table 2). In 2010 this percentage significantly increased to 36.0%, 662 of 1845 publications, (p<0.01; two tailed, paired t-test comparing the percent of international collaborations by country for 2000 and 2010). The number of countries represented grew from 35 in 2000 to 47 in 2010.

**Table 2:** Summary of publications analyzed for 2000 and 2010.

	2000	2010
Total number of publications	969	1845
Single country publications	766	1181
International publications*	203	664
Percent of international publications	20.9	36.0
Number of international partnerships**	158	381
Total number of countries in internationalpublications	35	47

* International publications were defined as those with authors from at least two different countries.

** International partnerships refer to country partnerships e.g. UK and US, not specific partnerships between lab A and lab B.

It is important to note that there are some differences between the two sets of journals for 2000 and 2010, as many journals prominent in 2010 were not yet in existence in 2000 (Table S1 and S2). Of the top 50 journals for both years, 26 journals are present are both lists, 20 journals in 2010 did not publish stem cell research in 2000 or were not in existence, and 4 journals on the 2010 list did not have high enough impact factors in 2000 to make the top 50 list. These differences are likely due to the large growth that stem cell research experienced from 2000 to 2010, as well as the growth of scientific journals overall. And on average, the journal impact factors were higher for 2010, at 15.77 versus 11.37 for 2000, possibly indicating an increase in the prominence of stem cells in general.

We also conducted a keyword analysis to determine changes within the field between 2000 and 2010. Over 9,000 keywords were associated with articles from 2000 and 15,000 with articles from 2010– the majority of which were unrelated to the specific types of stem cells analyzed. Only 17% and 19% respectively were related to stem cells in general (Table 1). The percentage of keywords related to both cancer stem cells and iPSCs saw an increase from 3.6% to 5.9% and 0% to 0.18% respectively (Table 1). This percentage remained relatively constant in the field of ESCs (1.7% to 1.6%) and decreased slightly for ASCs (8.1% to 6.3%). This growth may also account for some of the large differences in impact factors for certain journals in the dataset. Journals likely to publish cancer stem cell research, for instance, including *Breast Cancer Research and Treatment, Journal of Clinical Oncology,* and *Cancer Cell,* saw large increases in impact factor.

While the United States remained the most dominate country (with nearly 60% of total papers in both years), the most relative growth was exhibited by Asian countries, particularly China, whose percent of total stem cell papers examined increased from 0.41% to 4.61% (Table 3). There was also some modest relative growth in European countries, primarily Spain (3.41 to 4.28%), Italy (4.02 to 6.50%), the United Kingdom (9.39 to 14.6%) and Germany (7.33 to 10.8%). And while most European countries also saw a modest increase in their percentage of international stem cell publications, the United Kingdom experienced more substantial growth in this category, from 9.21% to 28.8%. Elsewhere in the world, despite recent increased investment in S&E research, nations in the Middle East as well as Central and South America have yet to publish substantially in top journals (aside from Israel).

**Table 3:** Percentage of the total number of publications and of international publications for 2000 and 2010 by country.* †

	2000		2010	
	% Total Papers	% Intl Papers	% Total Papers	% Intl Papers
**North America**	**66.4**	**79.3**	**65.6**	**75.9**
Canada	6.71	14.8	6.50	12.8
USA	59.7	64.5	59.1	63.1
**Asia**	**11.6**	**24.15**	**18.6**	**28.6**
China	0.41	0.99	4.61	7.22
India	0.31	0.49	0.16	0.15
Japan	10.0	20.7	9.05	11.6
Singapore	0.21	0.49	1.79	4.22
South Korea	0.31	1.48	2.11	3.77
Taiwan	0.31	0	0.87	1.66
**Europe**	**36.0**	**72.2**	**48.3**	**96.4**
France	8.36	19.2	6.99	13.4
Germany	7.33	19.2	10.8	22.6
Italy	4.02	9.36	6.50	11.9
Netherlands	3.51	9.85	5.14	10.5
Spain	3.41	5.42	4.28	9.19
United Kingdom	9.39	9.21	14.6	28.8
**Middle East**	**1.55**	**4.93**	**3.60**	**4.52**
Israel	1.55	4.93	3.47	3.92
Iran	0	0	0.16	0.15
Lebanon	0	0	<0.01	0.15
Saudi Arabia	0	0	0.11	0.3
**Latin America**	**0.52**	**1.47**	**0.38**	**1.20**
Brazil	0.10	0.49	0.38	1.05
Chile	0.21	0.49	<0.01	0.15
Mexico	0.21	0.99	0	0

* Only countries with a strong history of biomedical research or are relatively new entrants were selected for the table.

† Regional percentages (1) only take into account the countries represented in the table, and (2) sum to over 100%, as publications often have multiple authors. For example, a publication with authors in US, China and UK would be represented in all three countries.

For both 2000 and 2010, the highest number of publications resulted from two country collaborations, with the exception of the US-UK-Canada network in 2010 (Table 4). Many of the same collaborations that were prolific in publishing persisted from 2000 to 2010, with the majority of these collaborations involving the United States. In 2000, the most frequent collaborations were between the United States and Japan, resulting in 28 publications. While this partnership was still strong in 2010, with a total of 25 publications, both China and the United Kingdom collaborated more frequently with the United States in 2010 with 33 and 35 publications respectively. Furthermore, it is worth noting that while neither China nor South Korea made the top 20 collaborations list for 2000, both appeared as corresponding and secondary authors in 2010.

**Table 4:** Top 20 collaborations by publication for 2000 and 2010.

2000	# of Papers	2010	# of Papers
USA*-Japan	18	USA*-China	26
Japan*-USA	10	USA*-United Kingdom	20
Germany*-USA	9	United Kingdom*-USA	15
Canada*-USA	8	USA*-Canada	15
Italy*-USA	8	USA*-Germany	15
USA*-Canada	8	USA*-Japan	13
USA*-Germany	7	Japan*-USA	12
Germany*-France	6	Canada*-USA	10
Italy*-United Kingdom	6	France*-USA	9
USA*-France	6	Italy*-USA	9
USA*-United Kingdom	6	USA*-Italy	8
France*-United Kingdom	5	Australia*-USA	7
France*-USA	5	China*-USA	7
Israel*-USA	5	Germany*-USA	7
Netherlands*-USA	5	USA*-Canada-United Kingdom	7
United Kingdom*-USA	5	USA*-France	7
USA*-Spain	5	USA*-Netherlands	7
Germany*-Spain	4	USA*-South Korea	7
Italy*-Germany	4	USA*-Spain	7
Netherlands*-Spain	4	South Korea*-USA	6
Netherlands*-United Kingdom	4	Spain*-United Kingdom	6
Sweden*-Switzerland	4	Spain*-USA	6
United Kingdom*-USA	4	USA*-Taiwan	6
United Kingdom*-Germany	4		
USA*-Netherlands	4		
USA*-Switzerland	4		

* country with corresponding author.

Examining the list of corresponding authors from all publications, including single country publications and those resulting from international collaborations, again saw the United States leading (Table 5). In addition, while China only published twice in 2000 (tied for 18^th^ out of 28 countries) as a corresponding author, it rose to 8^th^ in 2010 with 51 publications.

**Table 5:** List of corresponding author countries for 2000 and 2010.

2000	# of Papers	2010	# of Papers
USA	505	USA	911
Japan	64	United Kingdom	145
United Kingdom	57	Japan	115
France	48	Germany	100
Canada	43	France	77
Germany	40	Italy	71
Italy	27	Canada	65
Netherlands	19	China	51
Australia	14	Netherlands	45
Spain	13	Spain	39
Israel	10	Australia	38
Switzerland	9	Switzerland	30
Austria	8	South Korea	24
Sweden	8	Sweden	24
Belgium	7	Belgium	22
Finland	3	Israel	19
Taiwan	3	Austria	15
China	2	Singapore	14
Denmark	2	Denmark	9
India	2	Taiwan	7
Brazil	1	Greece	4
Chile	1	India	3
Ireland	1	Ireland	3
Mexico	1	Czech Republic	3
Norway	1	Finland	2
Russia	1	Iran	2
Singapore	1	Norway	2
South Korea	1	Portugal	2
		Lebanon	1
		New Zealand	1
		Poland	1

Additionally, the number of publications with five or more different authoring countries increased from just five in 2000 (2.5% of international publications) to 38 in 2010 (5.7% of international collaborations). The publication featuring the largest number of distinct countries in 2000 involved 11 different countries (France-Australia-Belgium-Brazil-Denmark-United Kingdom-Hungary-Italy-Netherlands-Spain-Sweden), while the publication featuring the largest number of authoring countries in 2010 involved 15 different authors (Austria-Belgium-Czech Republic-Denmark-United Kingdom-France-Greece-Hungary-Israel-Norway-Poland-Portugal-Spain-Sweden-Switzerland). Consequently, while partnerships between two nations still dominate the number of publications produced, it does appear that more publications are originating from increasingly larger cross-border collaborations involving five or more countries.

**Figure 1:**
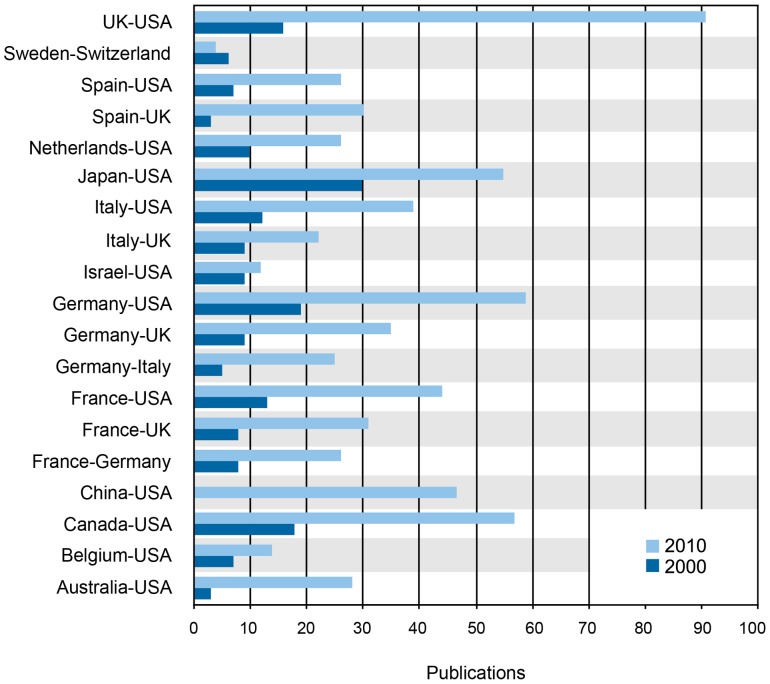
Top Collaborative Partnerships Based on Absolute Publication Counts. The top 15 collaborative partnerships were determined for each two-country pair in 2000 (dark blue) and 2010 (light blue). Ten partnerships rated high in both years: Canada-USA, France-Germany, France-UK, France-USA, Germany-UK, Germany-USA, Italy-USA, Japan-USA, Spain-USA, UK-USA. Overall, the number of publication increased for all top pairs with the exceptions of Sweden-Switzerland.

Next, the tendency of two nations to partner, whether one is a corresponding author or both are secondary authors on a publication, was determined. First we reviewed total partnership based on the absolute number of publications (Figure 1). Consistent with other results, US partnerships dominated the top 10 from 2000 and 2010. Furthermore, all collaborative partnership publications increased between 2000 and 2010, with the exception of Sweden-Switzerland partnership.

**Figure 2:**
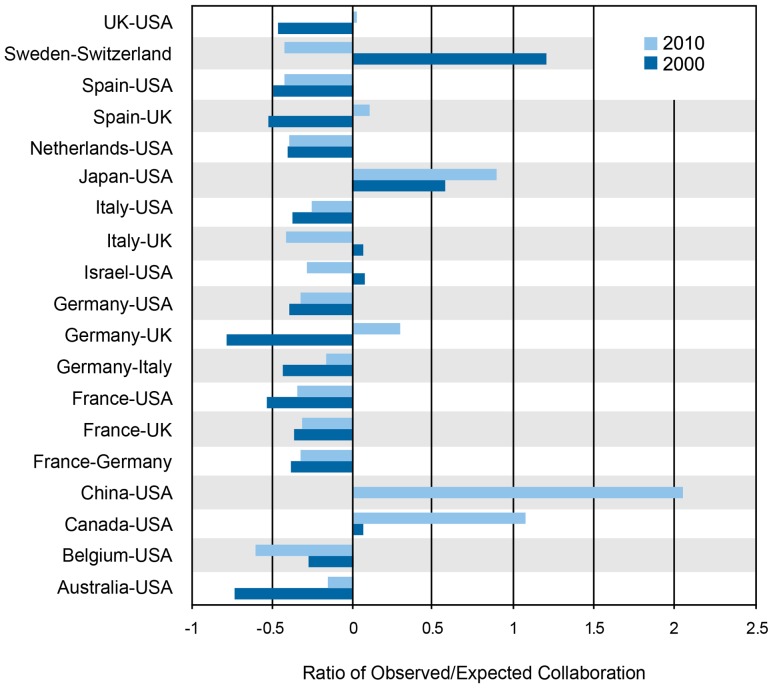
Observed Versus Expected Collaboration Patterns. The tendency of two nations to collaborate was determined by calculating a ratio of the observed to expected co-authorship frequency. A positive number illustrates unusually high tendency between two countries to collaborate with each other – in the context of their collaborations with other countries and the entire pool of collaborations– and a negative number reflects a lower than expected collaboration rate. The ratios from 2000 (dark blue) and 2010 (light blue) for top collaborative partnerships that existed both years and exhibited a positive value in one or both years are shown alongside each other.

Finally, the tendency of two nations to partner were analyzed by calculating a ratio of the observed to expected co-authorship frequency, E_r_ [8]. Positive values indicate a higher tendency to collaborate based on both nations’ tendencies to collaborate internationally. Of the top partnerships based on publications, only five in 2000 and six in 2010 yielded positive values (Figure 2 and Table S4). Only two positive partnerships were present in both years: Canada-US and Japan-US.

Overall, partnerships yielding positive values in 2000 and 2010 included: (1) countries that are geographically proximate, (2) countries that have a history of strong political and economic ties, or (3) partnerships in which one or both countries publish infrequently and primarily with one other country. An example of the third scenario is India and New Zealand, with an E*_r_* value of 46.33. Due to the nature of the equation used to calculate the ratio, partnerships such as India and New Zealand, in which both countries only published three times each, sharing 1 publication, yield large positive E*_r_* values. While many of the positive and high E*_r_* values can be explained by this phenomenon, some partnerships, such as the United States and Canada, the United States and Japan, Denmark and Finland, Italy and the United Kingdom, and Sweden and Switzerland exhibit positive E*_r_* values as these countries are likely to have an increased tendency to collaborate. While it is not possible to identify the exact reasons why these nations have a higher tendency to collaborate with each other, many of these partnerships feature countries that share geographic borders, economic and political ties (such as joint membership in the European Union), and or historical ties (such as the United States and Japan from post-World War II reconstruction).

Interestingly, the partnerships resulting in the most published papers for both 2000 and 2010, such as Germany-US, either scored values around zero or below it (Table S4, Figure 1 and 2). This indicates that, while these partnerships result in a high number of absolute papers published, this high productivity does not equate to a higher tendency for the nations to collaborate (Figure 2). The exceptions to this, however, include the United States with China (2.06, 5^th^ in number of publications 2010) and Japan (0.9, 4^th^ in number of publications 2010). This could be due to the growth of stem cell research in Asia. As emerging countries invest in the necessary personnel and infrastructure for biomedical research, it is probable that they would seek to engage in collaborations with countries with established histories in biomedical research. The overall doubling of international partnerships, from 158 in 2000 to 381 in 2010 is also noteworthy (Table 2).

## Discussion

As S&E research globalizes, it is increasingly important to understand the exact nature of international connections. Previous scholarship has demonstrated that one of the benefits resulting from international, collaborative research is a higher citation rate than work produced by scientists working at a single institution or within a single country [2]–[5]. Many possible explanations for this phenomenon, including the sharing of resources, ideas, and expertise, have been proposed. While it has also been suggested that increased collaboration lends to a larger number of self-citations, thus inflating citation rates, studies have indicated that this alone cannot explain the higher citation rates resulting from international collaborations [31]–[34]. Furthermore, the proportion of self-citations has remained relatively stable, around 10% of publications in the natural sciences, while the rate of international collaborations has increased, demonstrating the benefit of international collaborations in this area of research [14].

This study examined whether more countries were engaging in international collaborative research efforts or whether research primarily remained within national borders by mapping collaborative partnerships in the area of stem cell research. Stem cell research provided an ideal area of study due to its international nature and the existence of diverse national policies ranging from prohibitive to liberal [23]. And the promise of stem cell therapies for debilitating diseases such as macular degeneration, diabetes, and Parkinson’s disease has convinced many countries to embrace this area of research [35]. Indeed, from 2007–2011, the number of original research papers utilizing stem cells has more than doubled, demonstrating a growing global interest in this area [17].

From an analysis of 2,814 publications from 2000 and 2010, the United States was identified as the most prolific country in the field – accounting for nearly 60% of the articles from both years. While many emerging economies investing in S&E, including those in the Middle East and South America, have yet to become a substantial presence in the global stem cell research arena, this study indicates that Asian countries are publishing an increasing proportion of stem cell papers in top journals. This section will highlight three major themes. First, we will examine the growth of stem cell research in Asia, specifically in China. Then, we will discuss the continuing dominance of the United States and Europe in publications in high impact journals. Finally, we will comment on the persistence of traditional collaborative networks amidst the globalization of stem cell science.

### Stem Cell Research Leaders in Asia

Asia has exhibited the largest relative growth in this area of research between 2000 and 2010, with China playing a significant role (Table 2). The percentage of Chinese-authored stem cell papers in the top 50 journals grew more than ten-fold, while the percent of Chinese-authored international papers increased over seven times from 2000 to 2010. In contrast, relative growth rates in the United States, Canada and European countries remained more modest, with the exception of the United Kingdom, which saw more substantial increases, particularly in its share of international publications.

These trends are congruent with the general trends in S&E article output [36]. The number of publications worldwide increased at an annual rate of 2.6% between 1999 and 2009, with the highest average annual rates from Asian countries including China (16% annual increase), as opposed to the United States (1% annual increase) [36]. Consequently, in 2007, China surpassed the United Kingdom, Germany, and Japan to become the world’s second most prolific producer of publications, up from 14^th^ place in 1995 [36].

This phenomenon is consistent in stem cell publications from 2010 if all the publications are examined (Table S3). However, when only publications from the top 50 journals are considered, China still lags behind the United States, Canada, Japan, and European nations. While China produced 11.7% of all stem cell publications, it authored only 4.6% of publications in the top 50 journals. This indicates that while S&E research is growingly rapidly in China, much of the research has yet to translate into publications in high impact journals.

These increased publication rates are likely the result of recent growth in research and development (R&D) expenditures in Asian countries coupled with their permissive approaches to stem cell research. In the past decade, R&D expenditures have increased most dramatically in China, averaging about 19% annually, placing China third with 9% of the world’s R&D expenditures [36]. The ratio of R&D to GDP has also increased substantially during this time period for China. China’s ratio remains low, at 1.7%, but this number has more than doubled from 0.8% in 1999 [36].

Stem cell research in particular has enjoyed immense governmental support in China under the Ministry of Science and Technology’s (MOST) 1986 applied research initiative and its 1997 basic research program, known as the 863 and 973 plans, respectively (http://program.most.gov.cn). One factor that many researchers have identified as propelling China’s stem cell research forward is its permissive stem cell regulations [24], [37]–[40]. Therapeutic cloning, the use of surplus embryos from abortions, and the use of embryos created from somatic cell nuclear transfer, are all permitted [24]. In 2003, in response to concerns that more specific regulation was needed to oversee the growing area of research in China, the Ministry of Health and MOST jointly supported the ‘Ethical Guiding Principles on Human Embryonic Stem Cell Research’, which highlights the principles of respect for human life, informed consent, and safety [41]. The document proposes standardized procedures, institutional review boards, and professional qualifications for those working in the field of human ESC research, and it also bans any form of embryo or fetal tissue trade [41]. While these trends are promising, many safety and ethical concerns remain, particularly with regards to the safety of currently offered stem cell therapies [25].

### United States and Europe Retain their Dominance in Top Journals

While the relative growth rates in the number of publications from the United States were much lower than those in Asian countries, US S&E publications still remain some of the most cited worldwide. But in the decade between 1998 and 2008, the total share of the United States’ citations decreased from 45% to 36% [42]. Yet, the percentage of US-authored articles with the highest number of citations and appearing in top journals has remained relatively constant. The index of highly cited articles declined slightly from 1998 and 2008 from 1.83 to 1.78, but is still well above the expected index value of 1 (indexes of the European Union, China, and Japan are all below one) [36]. In 2010, while US articles were 28% of all articles in the cited period, they represented 49% of the top 1% of all cited articles [42]. And US authors were cited 76% more frequently than expected based on their percentage of publications worldwide in 2010 [42]. And in terms of R&D expenditures, the United States still leads the field, accounting for 31% of worldwide research expenditures [36].

This phenomenon is also evident in stem cell science. While the United States accounts for 40% of all stem cell publications in 2010 (down from about 43% in 2000 and 50% in 1995), it is responsible for nearly 60% of the papers analyzed from the top 50 journals by impact factor in 2010 (Table S3). Admittedly, some of this dominance in high impact journals may be the result of a familiarity with publication criteria and procedures, as well as more established reputations of researchers in the United States. However, the peer review process for top journals remains rigorous, thus reinforcing the prominence of US publications.

Like the United States, the European Union’s share of S&E publications decreased in the past decade; however, European authors have improved on citations per publication. According to the US National Science and Engineering Indicators, EU authored articles are cited less frequently than expected, although this measurement is improving from 27% less frequently in 2000 to only 6% less frequently in 2010 [43]. In stem cell research, European countries are still well represented in top journals. From 2000 to 2010, Germany, Italy, Netherlands, Spain and the United Kingdom all exhibited a relative increase of around 2–5% in their share of total stem cell publications. Furthermore, many of these European countries exhibited an even larger relative growth in percent of international publications, particularly the United Kingdom from 9.21 to 28.8%. Furthermore, France (4.9% of total publications, 7% of publications from top 50 journals), Germany (9.9%, 11%), Italy (5.7%, 6.5%), Netherlands (3.1%, 5.1%), Spain (3%, 4.3%), and the United Kingdom (7%, 14.6%) all have a higher percentage of publications in the top 50 journals analyzed than in the entire population of stem cell journals for 2010. These nations, thus, all produce a higher proportion of high impact publications relative to their proportion of all stem cell publications.

### Traditional Networks of Collaboration Remain Dominant Amidst Globalization

Examination of the top 20 collaborations, top 20 partnerships, and the country partnerships with a positive ratio of observed-to-expected co-authorship frequency (E*_r_*) reveals that collaborative partnerships correlate most strongly with traditional factors including geographic proximity (neighboring nations or those in the same regional supranational organizations), similarity in cultural practices and language, availability of funding, and shared histories amongst others (Table 3, Table S4, Figure 1 and Figure 2).

All the top 20 collaborations in 2000 involved either: (1) European nations, (2) United States and European nations, (3) Canada and European nations, (4) United States and Japan, (5) United States and Canada, or (6) United States and Israel (Table 3). The top 20 collaborations in 2010 also exhibited similar tendencies, with the United States involved in all of the top 20 collaborations.

The only difference between the 2000 and 2010 datasets was the appearance of collaborations between the United States and China, as well as the United States and South Korea in 2010. These collaborations could have evolved for multiple reasons, with the likely rationale the large investments in research made by both China and South Korea. China has employed many programs attracting Chinese national scientists who trained in the United States [43]. Those who do return to China and establish laboratories bring with them, across the Pacific, relationships with American researchers. This phenomenon also likely explains the wide range of institutions with which Chinese researchers collaborate, ranging from universities to private corporations. Analyzing the Chinese institutions with the highest number of international publications in 2010 and their partner institutions, no particular partnership appeared more than a handful of times.

The increased tendency for China and the United States to collaborate is further supported by the highest positive (2.1) ratio of observed-to-expected co-authorship frequency (E*_r_*) among the top partnerships for both 2000 and 2010 (Figures 1 and 2). It was one of only four partnerships that exhibited a shift, from 2000 to 2010, from a negative or zero E*_r_* value to a positive one. The other three partnerships include Germany-UK, Spain-UK, and UK-US. This increased tendency between European nations to collaborate could be the result of European Union related research programs. In 2000, the European Union established the European Research Area to unify research efforts among its members [44]. And under both its Sixth and Seventh Framework Programs, covering 2002–2013, a large portion of EU research funding specifically targets collaborative projects [45].

The increased tendency for the United Kingdom to collaborate internationally is also likely a result of various national initiatives, recognizing the importance of international collaboration and knowledge sharing, undertaken in the past decade. Some examples of these initiatives include the establishment of the International Stem Cell Forum (ISCF) by the UK Medical Research Council (MRC) along with eight other international funding agencies in 2003 to encourage international collaboration and funding efforts [46]. In 2008, the International Stem Cell Banking Initiative, led by the UK Stem Cell Bank to develop a global network of existing banks, was inaugurated [47]. Also in that year, the United Kingdom announced international collaborations with the California Institute for Regenerative Medicine (CIRM) with the focus of advancing stem cell therapies [48].

Due to the controversial nature of stem cell research, particularly human ESC research, the regulatory approaches taken by individual countries vary greatly and differ in degree from highly restrictive to highly permissive. Applying the definitions of restrictive, intermediate, and liberal stem cell policies from Isasi and Knoppers, no salient patterns can be observed between the top 20 collaborations or partnerships with their national stem cell policies [49]. All policy combinations–restrictive-restrictive, restrictive-intermediate, restrictive-liberal, intermediate-intermediate, intermediate-liberal, and liberal-liberal–can be found. This is consistent with other recent studies examining the use of human ESC lines in international research under differing policy regimes. These studies have concluded that innovation driven rather than policy driven considerations have been most influential in the decision to use various human ESC lines [50], [51].

It is important to emphasize that this study does not try to prove any type of causal relationship between various factors such as policy, geography, economic ties, shared histories, and cultural similarities with the formation of research collaborations. Many factors influence research patterns, and it is interesting to examine any correlations between these factors and the extant patterns in collaboration; however, no causation is implied by the study.

## Conclusion

Scientists decide to collaborate internationally for various reasons, and multiple factors play a role in their decision. The availability of a cell line, funding opportunities, prior collaborative or personal experiences, institutional partnerships, geographic proximity, language, and cultural similarities are just some of the possible factors. As a study examining the broader trends in the evolution of international collaborative research networks, this study cannot provide a detailed examination into the nature and rationale for each collaborative effort.

Furthermore, journal requirements also vary greatly in their definition of authorship, thus some listed authors/collaborators may only have played a very small role in the research, such as sharing a biological reagent or editing the final paper, while others may have been more involved. This study, thus, is also unable to identify the degree to which nations are collaborating. And while the increased prevalence of cancer stem cell and iPSC research may admittedly skew the countries publishing in the top 50 journals, as certain countries may have more programs invested in these areas (e.g. Japan and iPSCs), further analysis in the future will be required to measure the extent of the skew, if any exists. Further research is also required to better quantify the benefits of participating in an international research network from a citation perspective. This will contribute to our understanding of the motivations for engaging in international collaborations, the rationales behind selecting collaborators, and the extent and nature of international collaborations in an effort to promote more cross-border knowledge sharing and research pursuits.

Regardless, this study is among the first of its kind to examine the global networks of collaboration and their evolution in stem cell science. We found that over the past decade, collaborative relationships have increased, both in absolute number of publications and as a percentage of total stem cell publications, involving scientists from 47 nations (up from 28).

While scientists in North America and Europe, with strong traditions of biomedical research, continue to command a large percentage of publications in high impact journals, more recent entrants to the field, particularly those from Asian countries, are beginning to gain a larger portion of these publications. They have done so not only by increasing their investment in R&D and supporting internal research efforts, but also by reaching out to the international community and establishing partnerships with scientists and institutions from other nations. We believe that this trend will persist in the future, evident from the many partnerships from publications from 2010 that were not present from an examination of publications from 2000. This development could potentially lead to increased relationship building with new partners in regions such as Latin America and the Middle East, which are just beginning to encourage stem cell research. As stem cell research continues to grow as a highly collaborative field of research, it will likely provide more insight on cross-border collaboration in research and call for increased international stem cell policy regimes to facilitate future collaborations.

## Supporting Information

Table S1 Top 50 journals by JCR 2000 impact factor and the number of stem cell articles collected from each journal.
(DOCX)

Table S2 Top 50 journals by JCR 2010 impact factor and the number of stem cell articles collected from each journal.
(DOCX)

Table S3 Thomson Reuters’ ISI Knowledge Web of Science Top 20 countries based on number of publications from 2000 and 2010.
(DOCX)

Table S4 Top partnerships by number of publications for 2000 and 2010. The countries in the partnerships can either both be secondary authors or one of them the corresponding author. Partnerships with fewer than 10 publications were not included in the table.
(DOCX)
