# Supplementary material for: Globalization of Stem Cell Science: An Examination of Current and Past Collaborative Research Networks
Source: PLoS One. 2013 Sep 12;8(9):e73598. doi: 10.1371/journal.pone.0073598 (PMC3772010; doi:10.1371/journal.pone.0073598)
Supplement: Table S1 — Top 50 journals by JCR 2000 impact factor and the number of stem cell articles collected from each journal. (DOCX) [file pone.0073598.s001.docx]

**Table S1 –** Top 50 journals by JCR 2000 impact factor and the number of stem cell articles collected from each journal.

| **Journal Title** | **# Articles in 2000** | **2000 Impact Factor** |
| --- | --- | --- |
| Cell | 12 | 32.44 |
| Nature Genetics | 17 | 30.91 |
| New England Journal of Medicine | 7 | 29.512 |
| Nature Medicine | 13 | 27.905 |
| Nature | 18 | 25.814 |
| Science | 19 | 23.872 |
| Immunity | 11 | 21.083 |
| Molecular Cell | 3 | 18.195 |
| Journal of Experimental Medicine | 19 | 15.236 |
| Neuron | 12 | 15.081 |
| EMBO Journal | 16 | 13.999 |
| Journal of Cell Biology | 13 | 13.955 |
| Nature Neuroscience | 3 | 12.636 |
| Journal of Clinical Investigation | 19 | 12.015 |
| Nature Biotechnology | 10 | 11.542 |
| Plant Cell | 3 | 11.093 |
| Proceedings of the National Academy of Science USA | 68 | 10.789 |
| Lancet | 12 | 10.232 |
| Molecular and Cellular Biology | 37 | 9.66 |
| Development | 40 | 9.353 |
| FASEB Journal | 4 | 9.249 |
| Circulation Research | 4 | 9.193 |
| Human Molecular Genetics | 11 | 9.048 |
| Blood | 210 | 8.977 |
| Journal of Clinical Oncology | 29 | 8.773 |
| Journal of Neuroscience | 33 | 8.502 |
| Molecular Biology of the Cell | 4 | 8.482 |
| Cancer Research | 19 | 8.46 |
| Current Biology | 12 | 8.393 |
| Diabetes | 4 | 7.715 |
| Genome Research | 3 | 7.615 |
| Journal of Biological Chemistry | 50 | 7.368 |
| Hepatology | 8 | 7.304 |
| American Journal of Pathology | 16 | 6.971 |
| Arthritis and Rheumatism | 5 | 6.841 |
| Journal of Immunology | 26 | 6.834 |
| Human Gene Therapy | 28 | 6.796 |
| Oncogene | 25 | 6.49 |
| Molecular Microbiology | 2 | 6.339 |
| Molecular Endocrinology | 4 | 6.251 |
| Journal of Cell Science | 14 | 5.996 |
| Gene Therapy | 25 | 5.964 |
| Journal of Virology | 28 | 5.93 |
| Journal of Cerebral Blood Flow and Metabolism | 2 | 5.926 |
| Journal of Bone and Mineral Research | 3 | 5.877 |
| Journal of the American Society of Nephrology | 4 | 5.745 |
| Molecular Pharmacology | 4 | 5.678 |
| Journal of Neuropathology and Experimental Neurology | 2 | 5.565 |
| Developmental Biology | 23 | 5.54 |
| Journal of Molecular Biology | 5 | 5.338 |
