# Supplementary material for: Globalization of Stem Cell Science: An Examination of Current and Past Collaborative Research Networks
Source: PLoS One. 2013 Sep 12;8(9):e73598. doi: 10.1371/journal.pone.0073598 (PMC3772010; doi:10.1371/journal.pone.0073598)
Supplement: Table S2 — Top 50 journals by JCR 2010 impact factor and the number of stem cell articles collected from each journal. (DOCX) [file pone.0073598.s002.docx]

**Table S2 –** Top 50 journals by JCR 2010 impact factor and the number of stem cell articles collected from each journal.

| **Journal Title** | **# Articles in 2010** | **2010 Impact Factor** |
| --- | --- | --- |
| New England Journal of Medicine | 14 | 53.486 |
| Nature Genetics | 9 | 36.33 |
| Nature | 75 | 36.104 |
| Cell | 38 | 32.406 |
| Science | 22 | 31.377 |
| Nature Biotechnology | 23 | 31.09 |
| Cancer Cell | 26 | 26.925 |
| Breast Cancer Research and Treatment | 93 | 25.943 |
| Nature Medicine | 19 | 25.43 |
| Immunity | 10 | 24.221 |
| Nature Cell Biology | 23 | 19.407 |
| Journal of Clinical Oncology | 54 | 18.97 |
| Lancet Oncology | 8 | 17.764 |
| Journal of Experimental Medicine | 15 | 14.776 |
| Journal of The American College of Cardiology | 8 | 14.293 |
| Molecular Cell | 13 | 14.194 |
| Nature Neuroscience | 14 | 14.191 |
| Journal of Clinical Investigation | 52 | 14.152 |
| Neuron | 10 | 14.027 |
| Developmental Cell | 29 | 13.946 |
| Nature Structural Molecular Biology | 12 | 13.685 |
| Genome Research | 22 | 13.588 |
| Current Opinion in Cell Biology | 13 | 13.54 |
| Journal of Molecular Biology | 7 | 13.4 |
| PLOS Biology | 17 | 12.472 |
| Hepatology | 21 | 10.885 |
| Advanced Materials | 10 | 10.88 |
| Gut | 10 | 10.618 |
| Blood | 292 | 10.558 |
| Embo Journal | 15 | 10.124 |
| Current Biology | 14 | 10.026 |
| Journal of Cell Biology | 20 | 9.921 |
| ACS Nano | 13 | 9.865 |
| Proceedings of the National Academy of Sciences (US) | 215 | 9.771 |
| European Cell Materials | 24 | 9.65 |
| PLOS Genetics | 32 | 9.543 |
| Circulation Research | 30 | 9.504 |
| Cell Research | 13 | 9.417 |
| Plant Cell | 15 | 9.396 |
| Journal of Allergy and Clinical Immunology | 12 | 9.273 |
| Brain | 11 | 9.232 |
| Cell Death and Differentiation | 14 | 9.05 |
| Leukemia | 53 | 8.966 |
| Diabetes | 15 | 8.889 |
| EMBO Molecular Medicine | 7 | 8.833 |
| Nature Protocols | 28 | 8.362 |
| Cancer Research | 113 | 8.234 |
| Human Molecular Genetics | 26 | 8.058 |
| Stem Cells | 199 | 7.871 |
