# Supplementary material for: Globalization of Stem Cell Science: An Examination of Current and Past Collaborative Research Networks
Source: PLoS One. 2013 Sep 12;8(9):e73598. doi: 10.1371/journal.pone.0073598 (PMC3772010; doi:10.1371/journal.pone.0073598)
Supplement: Table S3 — Thomson Reuters’ ISI Knowledge Web of Science Top 20 countries based on number of publications from 2000 and 2010. (DOCX) [file pone.0073598.s003.docx]

**Table S3 –** Thomson Reuters’ ISI Knowledge Web of Science Top 20 countries based on number of publications from 2000 and 2010*.

| **2010** |  |  |  | | **2000** | |  | |  | |
| --- | --- | --- | --- | --- | --- | --- | --- | --- | --- | --- |
| **Country** | **# of Papers** | **% of Total Publications** | | **Country** | | **# of Papers** | | **% of Total Publications** | |  |
| **USA** | 5632 | 39.0 |  | | USA | | 1694 | | 43.9 | |
| **China** | 1688 | 11.7 |  | | Japan | | 465 | | 12.0 | |
| **Germany** | 1420 | 9.8 |  | | Germany | | 392 | | 10.2 | |
| **Japan** | 1351 | 9.4 |  | | England | | 303 | | 7.9 | |
| **United Kingdom** | 998 | 6.9 |  | | France | | 271 | | 7.0 | |
| **Italy** | 832 | 5.8 |  | | Italy | | 217 | | 5.6 | |
| **France** | 720 | 5.0 |  | | Canada | | 215 | | 5.6 | |
| **Canada** | 668 | 4.6 |  | | Netherlands | | 126 | | 3.3 | |
| **South Korea** | 666 | 4.6 |  | | Spain | | 110 | | 2.9 | |
| **Netherlands** | 454 | 3.1 |  | | Australia | | 94 | | 2.4 | |
| **Spain** | 432 | 3.0 |  | | Sweden | | 88 | | 2.3 | |
| **Australia** | 400 | 2.8 |  | | Switzerland | | 77 | | 2.0 | |
| **Switzerland** | 335 | 2.3 |  | | Israel | | 60 | | 1.6 | |
| **Sweden** | 289 | 2.0 |  | | Belgium | | 58 | | 1.5 | |
| **Brazil** | 242 | 1.7 |  | | Austria | | 52 | | 1.3 | |
| **Taiwan** | 234 | 1.6 |  | | South Korea | | 47 | | 1.2 | |
| **Singapore** | 224 | 1.6 |  | | Scotland | | 45 | | 1.2 | |
| **Belgium** | 190 | 1.3 |  | | China | | 32 | | 0.8 | |
| **Israel** | 189 | 1.3 |  | | Finland | | 30 | | 0.8 | |
| **India** | 174 | 1.2 |  | | Denmark | | 28 | | 0.7 | |

*This analysis takes into account all the publications pertaining to stem cell research, not just those in the top 50 journals by impact factor.
