# Supplementary material for: Globalization of Stem Cell Science: An Examination of Current and Past Collaborative Research Networks
Source: PLoS One. 2013 Sep 12;8(9):e73598. doi: 10.1371/journal.pone.0073598 (PMC3772010; doi:10.1371/journal.pone.0073598)
Supplement: Table S4 — Top partnerships by number of publications for 2000 and 2010. The countries in the partnerships can either both be secondary authors or one of them the corresponding author. Partnerships with fewer than 10 publications were not included in the table. (DOCX) [file pone.0073598.s004.docx]

**Table S4** – Top partnerships by number of publications for 2000 and 2010. The countries in the partnerships can either both be secondary authors or one of them the corresponding author. Partnerships with fewer than 10 publications were not included in the table.

| **2000** |  |  | | **2010** | |  |
| --- | --- | --- | --- | --- | --- | --- |
| **Partnership** | **Number of Publications** |  | | **Partnership** | | **Number of Publications** |
| Japan-USA | 30 |  | United Kingdom-USA | | 93 | |
| Germany-USA | 19 |  | | Germany-USA | | 59 |
| Canada-USA | 18 |  | | Canada-USA | | 57 |
| United Kingdom-USA | 16 |  | | Japan-USA | | 55 |
| France-USA | 13 |  | | China-USA | | 46 |
| Italy-USA | 12 |  | | France-USA | | 44 |
|  |  |  | | Italy-USA | | 39 |
|  |  |  | | United Kingdom-Germany | | 35 |
|  |  |  | | United Kingdom-France | | 31 |
|  |  |  | | United Kingdom-Spain | | 30 |
|  |  |  | | Australia-USA | | 28 |
|  |  |  | | France-Germany | | 26 |
|  |  |  | | Netherlands-USA | | 26 |
|  |  |  | | Spain-USA | | 26 |
|  |  |  | | Germany-Italy | | 25 |
|  |  |  | | United Kingdom-Italy | | 22 |
|  |  |  | | United Kingdom-Netherlands | | 22 |
|  |  |  | | Germany-Netherlands | | 22 |
|  |  |  | | Sweden-USA | | 20 |
|  |  |  | | France-Italy | | 20 |
|  |  |  | | Canada-United Kingdom | | 19 |
|  |  |  | | South Korea-USA | | 19 |
|  |  |  | | Australia-United Kingdom | | 18 |
|  |  |  | | Belgium-France | | 18 |
|  |  |  | | Germany-Switzerland | | 17 |
|  |  |  | | Singapore-USA | | 17 |
|  |  |  | | Switzerland-USA | | 17 |
|  |  |  | | Belgium-Germany | | 16 |
|  |  |  | | Canada-Germany | | 15 |
|  |  |  | | United Kingdom-Japan | | 15 |
|  |  |  | | Germany-Spain | | 15 |
|  |  |  | | Belgium-USA | | 14 |
|  |  |  | | Czech Republic-Germany | | 13 |
|  |  |  | | France-Spain | | 13 |
|  |  |  | | Belgium-United Kingdom | | 12 |
|  |  |  | | United Kingdom-Sweden | | 12 |
|  |  |  | | United Kingdom-Switzerland | | 12 |
|  |  |  | | France-Switzerland | | 12 |
|  |  |  | | Israel-USA | | 12 |
|  |  |  | | Italy-Spain | | 12 |
|  |  |  | | Austria-Germany | | 11 |
|  |  |  | | Austria-USA | | 11 |
|  |  |  | | Germany-Sweden | | 11 |
|  |  |  | | France-Netherlands | | 10 |
|  |  |  | | France-Sweden | | 10 |
